# Photodynamic Therapy for the Treatment of Infected Leg Ulcers—A Pilot Study

**DOI:** 10.3390/antibiotics10050506

**Published:** 2021-04-29

**Authors:** Magdalena Krupka, Andrzej Bożek, Dorota Bartusik-Aebisher, Grzegorz Cieślar, Aleksandra Kawczyk-Krupka

**Affiliations:** 1 Clinical Department of Internal Medicine, Dermatology and Allergology, Medical University of Silesia, Katowice, M. Sklodowskiej-Curie 10, 41-800 Zabrze, Poland; magda.krupka94@gmail.com (M.K.); andrzej.bozek@sum.edu.pl (A.B.); 2 Department of Internal Medicine, Angiology and Physical Medicine, Centre for Laser Diagnostics and Therapy, Medical University of Silesia, Katowice, Batorego 15, 41-902 Bytom, Poland; cieslar1@tlen.pl; 3 Department of Biochemistry and General Chemistry, Medical College of the University of Rzeszów, University of Rzeszów, Kopisto 2A, 35-310 Rzeszów, Poland; dbartusik-aebisher@ur.edu.pl

**Keywords:** chronic leg ulcers, photodynamic therapy, 5-aminolevulinic acid

## Abstract

Chronic and infected leg ulcers (LUs) are painful, debilitating, resistant to antibiotics, and immensely reduce a patient’s quality of life. The purpose of our study was to demonstrate the efficacy of photodynamic therapy (PDT) for the treatment of infected chronic LUs. Patients were randomized into two experimental groups: the first group received 5-aminolevulinic acid photodynamic therapy (ALA-PDT) (10 patients), and the second group of 10 patients received local octenidine dihydrochloride (Octenilin gel) exposed to a placebo light source with an inserted filter that mimiced red light. In the PDT group, we used 20% ALA topically applied for 4 hrs and irradiation from a Diomed laser source with a wavelength of 630 nm at a fluency of 80 J/cm^2^. ALA-PDT was performed 10 times during a 14-day hospitalization in 10 patients of both sexes aged 40–85 years with chronic leg ulcers. Treatments were carried out at 3-week intervals for 3–5 cycles. At 8-month follow-up with the PDT group, complete remission (CR) was obtained in four patients (40%), partial response (>50% reduction in ulcer diameter) in four patients (40%), and no response in two patients (20%) who additionally developed deterioration of the local condition with swelling, erythema, and inflammation. To assess the degree of pain during the trials, we used a numeric rating scale (NRS). From the preliminary results obtained, we concluded that PDT can be used to treat leg ulcers as a minimally invasive and effective method with no serious side effects, although further studies on a larger group of patients with LUs are warranted.

## 1. Introduction

Chronic leg ulcers (LUs), also known as chronic lower limb ulcers, are chronic leg wounds that have no tendency to heal after 3 months of appropriate treatment, or are not fully healed after 12 months. The etiopathogenesis of leg ulcers is extremely complex, however, the most common cause of leg ulcers are diseases of the veins and arteries [1].

### 1.1. LUs—Epidemiology

A retrospective cohort study using THIN (The Health Improvement Network) data reported that in the UK, 53% of all venous LUs healed within 12 months, with a mean healing time of three months. Similar significant evidence–practice gaps have been reported worldwide, including reports from several developed countries [2]. Chronic LUs are a significant problem worldwide, affecting 1% of the adult population and occurring in 4% of people over 65 years of age [3]. The quality of life of patients with leg ulcers is very poor. This is due to pain as well as shame, embarrassment, and isolation, and LUs cause higher levels of depression and anxiety [4].

### 1.2. LUs—Etiopathogenesis

Most often, the cause of leg ulcers are disorders of the blood vessels, veins and arteries (85%) [1]. It is a problem with a complex etiopathogenesis, caused by diseases of the venous system, such as venous insufficiency, varicose veins, and previous deep vein thrombosis. Chronic venous insufficiency affects more than 35% of patients, and the development of leg ulcers is found in over 65% of all cases of this disease. A venous leg ulcer is the loss of full-thickness skin, usually around the medial ankle, that does not heal spontaneously and is sustained by existing disturbances in venous outflow. The current and constantly improved CEAP classification distinguishes two clinical categories of venous ulcers that may coexist: C5—the presence of a scar after a healed ulcer and C6—an active venous ulceration.

In 10% of cases, ulcers are caused by arterial diseases, and 10% of LUs are caused by the combined occurrence of chronic venous insufficiency (CVI) and peripheral arterial disease (PAD) with arterial stenosis. Five percent of cases are the result of diabetic polyneuropathy (neuropathic foot (NF)), often with concomitant limb arterial stenosis and diabetes. LUs are further characterized by infection, ulceration, or destruction of deep tissues of the foot (including bones) in diabetic patients, and the presence of neurological disorders and peripheral arterial disease in the lower limbs with varying degrees of advancement.

Due to the pathomechanism, there are several classes: neuropathic foot (the most common), ischemic foot, and neuropathic–ischemic foot. It is very important to distinguish the neuropathic foot from the ischemic foot, especially using angio CT and duplex Doppler assessment. Motor neuropathy leads to atrophy of the foot muscles, disrupting the cooperation of the extensors and flexors, and contractures. Sensory neuropathy (disturbances of the sensation of pain, temperature, and touch) exposes the affected patient to repeated uncontrolled injuries that lead to the formation of ulcers, as well as to changes due to autonomic neuropathy as in the formation of arteriovenous fistulas and trophic disorders.

Atherosclerosis of the arteries of the lower extremities leads to ischemia of the foot. Local osteoporosis develops along with associated osteomyelitis, sterile necrosis, fractures, joint dislocations, and, as a result, significant deformation of the foot may occur.

Two percent of leg ulcers are the result of injuries, and in the remaining cases, the origin of the ulcers may be the result of ulcerative skin cancer, abnormal wound healing after trauma, angiodysplasia, vascular ulcer, pyoderma gangrenosum, embolism caused by cholesterol, antiphospholipid syndrome, calciphylaxis in chronic renal failure, necrobiosis lipoidica, hematological and autoimmune diseases, and autoimmune bullous dermatoses.

In tropical countries, there is a paucity of epidemiological studies regarding the prevalence and etiology of leg ulcers. A study from one center in India suggests that leprosy (40%), diabetes (23%), venous disease (11%) and trauma (13%) are among the most common causes of leg injuries.

### 1.3. Symptoms of LUs

The most frequently used clinical signs of infected LUs are edema, malodor, erythema, increased ulcer pain, amplified exudate levels or purulent exudate, increased local temperature around the wound, delayed or non-healing, and friable granulation tissue. The main complaints of patients are burning, tingling, sharp pain, limb edema, loss of sensation, difficulty in walking, and musculoskeletal deformities.

The differential diagnosis of the most important causes of LUs is presented in Table 1.

### 1.4. LU Infection

Permanent bacterial colonization of wounds is an additional factor that significantly worsens the prognosis and extends the treatment process [5,6]. Since wound colonization is most frequently polymicrobial, involving numerous microorganisms that are potentially pathogenic, any wound is at some risk of becoming infected.

The microbial signs for infection are still controversial [7]. Some consider that the density of microorganisms is the critical factor in determining whether a wound is likely to heal; however, others suggest that the presence of specific pathogens is of primary importance in delayed healing. The most common pathological flora are formed by the following aerobic bacteria: *Staphylococcus aureus*, β-hemolytic *streptococcus*, *Streptococcus* spp. *(fecal)*, *Streptococcus* spp. *(viridans)*, *Corynebacterium xerosis*, *Corynebacterium* sp., *Bacillus* sp., *Escherichia coli, Escherichia hermanii, Serratia liquefaciens, Klebsiella pneumoniae, Klebsiella oxytoca, Enterobacter cloacae, Enterobacter aerogenes, Citrobacter freundii, Proteus mirabilis, Proteus vulgaris, Providencia stuartii, Morganella morganii, Acinetobacter calcoaceticus, Pseudomonas aeruginosa, Bacteroides stercoris and Fusobacterium necrophorum*. Common pathological anaerobic bacteria in LUs include: *Peptostreptococcus magnus*, *Clostridium perfringens*, *Clostridium septicum*, *Clostridium histolyticum*, *Clostridium difficile*, *Eubacterium limosum* and *Bacteroides uniformis*.

The clinical criteria indicating that infection is present are increased pain, an enlarging ulcer, cellulitis, and pyrexia [8,9,10]. Contributing to the enlargement of ulcers is the formation of oozing wounds, which increases the risk of complications, which also leads to a significant deterioration in the quality of life. In this era of increasing antibiotic resistance, it is important to search for new methods of therapy. Current research has demonstrated the effectiveness of antimicrobial photodynamic therapy (A-PDT) for a range of Gram-positive and Gram-negative bacteria, viruses and fungi, accelerating the healing of chronic wounds, and non-healing and recurrent lower leg ulcers.

### 1.5. LU Therapy

Chronic wound therapy, regardless of etiology, is a difficult, long, and demanding process requiring specialist involvement. Existing complementary methods available for ulcer treatment, such as compression therapy, surgery, endovascular treatment, antibiotic therapy, local treatment with the use of active dressings, as well as skin transplants, often do not bring any measurable therapeutic benefit. Therapeutic options include surgical or endovascular treatment; however, a successful treatment usually needs an interdisciplinary approach. In addition to compressive therapy, local treatment contains cleansing, debridement techniques, and dressings that diminish infection/colonization and simplify healing. Some systemic drugs can increase the rate of leg ulcer healing, such as synthetic flavonoids, pentoxifylline, sulodexide, cilostazol, and rivaroxaban [11].

### 1.6. PDT

Photodynamic therapy is a novel treatment method that works through the interaction of light of a specific wavelength, a photosensitizer, and oxygen. The mechanism of action of PDT is based on a cytotoxic, vascular, antimicrobial, and immunomodulating effect. PDT is successfully used as a radical or palliative treatment method in pre- and neoplastic diseases, but also in diseases of microbiological, autoimmune, and inflammatory etiology.

It was shown that PDT may become an alternative to antibiotic therapy, as, unlike classical antibiotic therapy, the development of microbial resistance mechanisms has not been reported to date using this treatment approach. It is also effective against multi-drug-resistant microorganisms with minimal adverse effects [12,13,14,15].

## 2. Results

**Figure 1:**
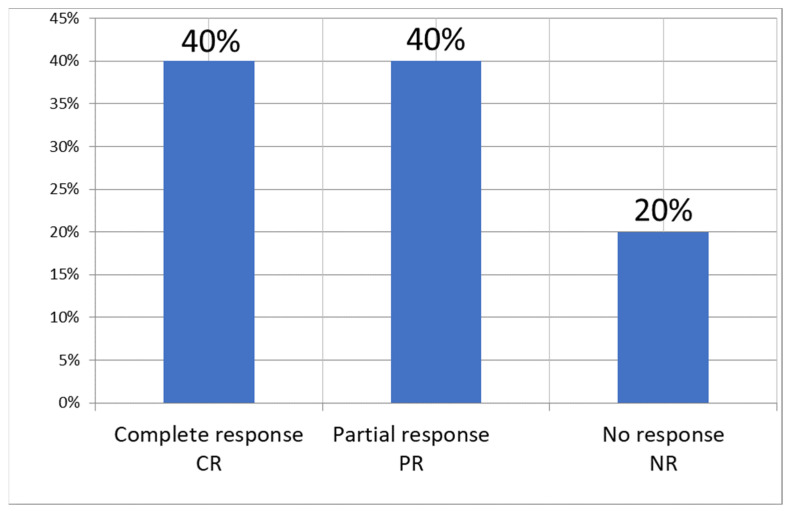
Response to photodynamic therapy in 10 patients with leg ulcers.

**Figure 2:**
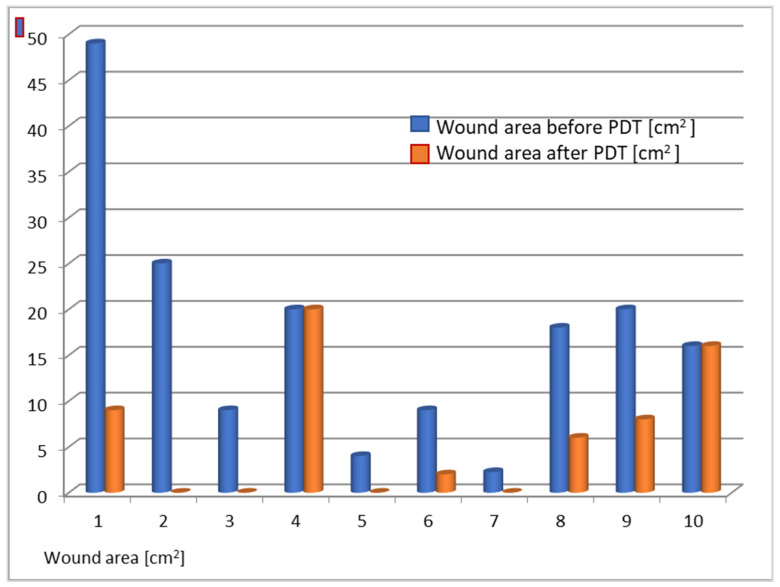
Wound area before (blue column) and after (orange column) PDT.

**Figure 3:**
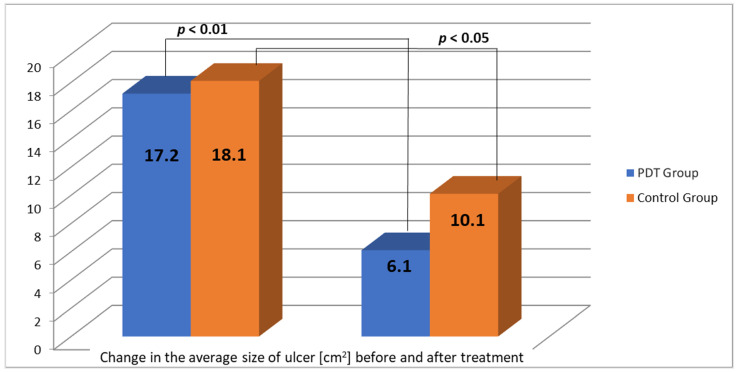
Change in the average size of ulcers before and after PDT in comparison to the control group.

After the series of treatments, wound size assessment, adverse effects, pain and tolerance of the procedure, microbiological cultures, and blood control tests were performed. At 8-month follow-up, complete remission (CR) was obtained in four patients (40%), partial response (>50% reduction in ulcer diameter) in four patients (40%). No response in two patients (20%) was observed with additional deterioration of the local condition, with swelling, erythema, and inflammation. Figure 1 and Table 2 present the obtained results. The wound size before and after PDT diminished and the changes are presented in Table 2 and Figure 1 and Figure 2. The wound area changes of each patient treated with PDT are presented in Figure 2. The average wound size decreased from 17.2 cm^2^ to 6.1 cm^2^ in comparison to the control group (18.1 cm^2^ to 8.1 cm^2^) (Figure 3).

### Adverse Reactions

The treatment was well tolerated, with moderate pain during treatment. Moderate localized edema and erythema were observed immediately after light treatment for no longer than 1–2 days post-treatment.

**Figure 4:**
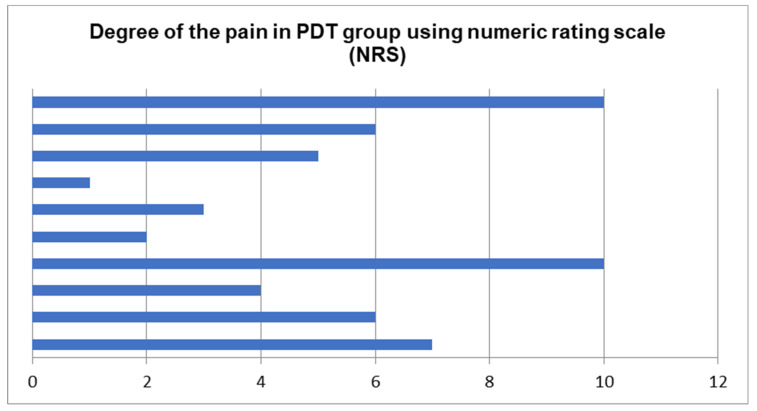
Degree of pain in each patient from the PDT group.

In experiments to assess the degree of pain, we used a Numeric Rating Scale (NRS). NRS is the simplest and most commonly used numeric scale in which the patient rates the pain from 0 (no pain) to 10 (worst pain).The degree of the pain present in each patient from the PDT group is presented in Figure 4.

Only in two patients (20%) was a deterioration of the local condition observed, with severe swelling, erythema, and inflammation. When the temperature of the end of the fiber optic cooled, the pain diminished.

**Figure 5:**
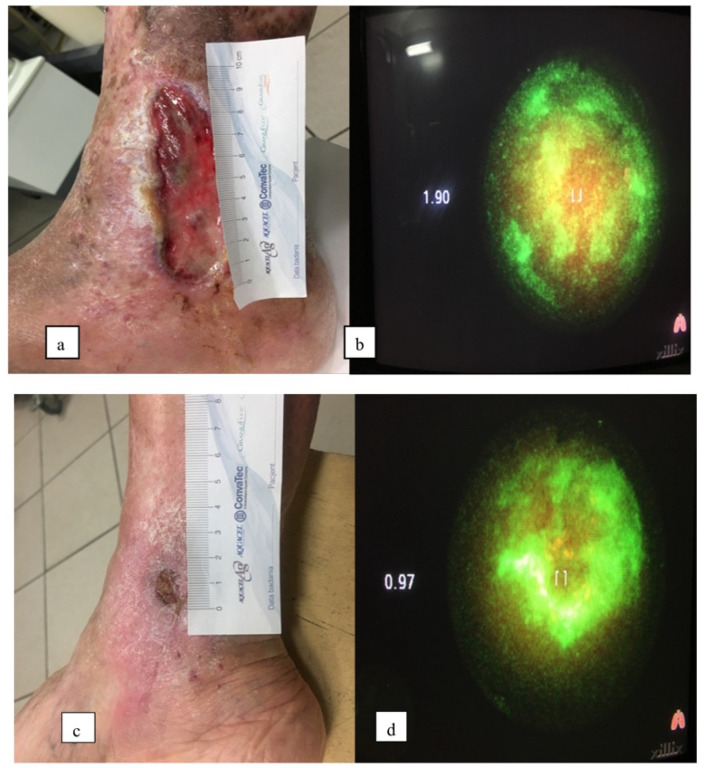
Patient 1 with leg ulcer in white light (**a**,**c**) and autofluorescent light (**b**,**d**) before (**a**,**b**) and after ALA/PDT (**c**,**d**).

**Figure 6:**
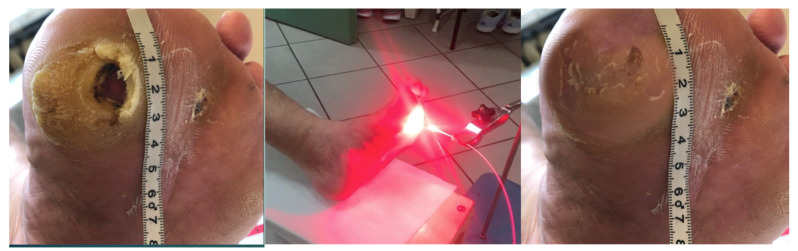
Patient 2 with foot ulcer after 3 cycles of PDT.

**Figure 7:**
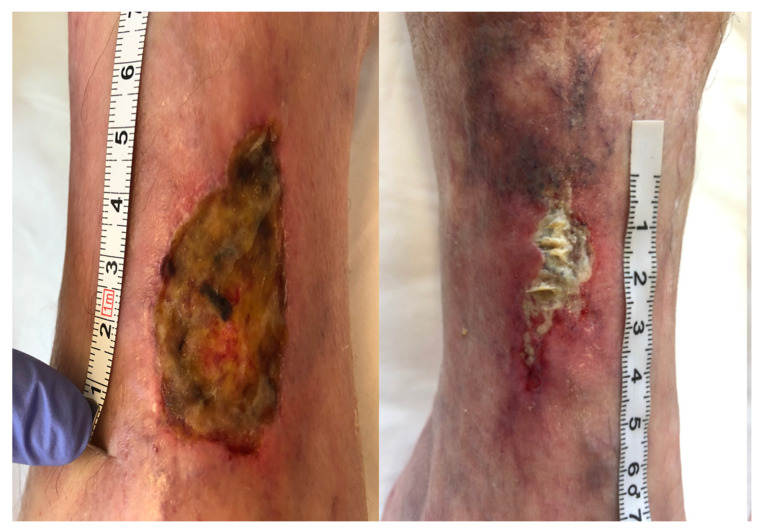
Patient 3 with foot ulcer after 3 cycles of PDT.

**Figure 8:**
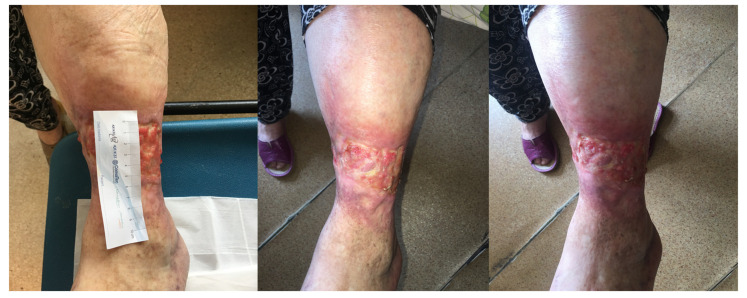
Patient 4 with leg ulcer before and after 3 cycles PDT.

**Figure 9:**
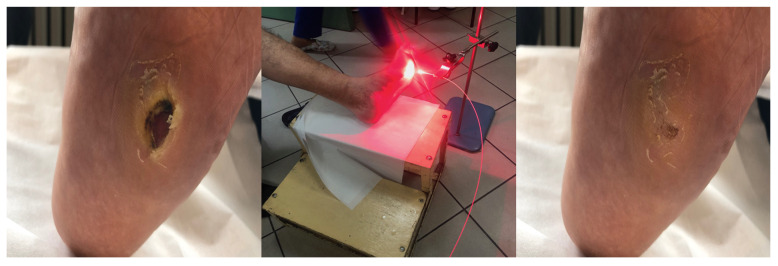
Patient 5 with foot ulcer before and after 2 cycles of PDT**.**

Figure 5, Figure 6, Figure 7, Figure 8 and Figure 9 present treated patients with leg ulcers before and after PDT.

## 3. Discussion

Our study is a pilot study, and, unfortunately, our clinical trials with photodynamic therapy for leg ulcers have been severely limited during the SARS-CoV-2 pandemic era.

Chronic leg ulcers are a significant global problem. Leg ulcers are a serious complication of chronic venous insufficiency, untreated varicose veins of the lower extremities, and deep vein thrombosis [15,16]. They are wounds that are difficult to heal. Treatment time is lengthy and sometimes requires several, or even a dozen, years, leading to many complications, e.g., cellulitis, joint deformities, step and limitation of its mobility, or even permanent disability. Research shows that the lack of proper treatment for chronic venous insufficiency can lead to ulcer spread and infection.Billions of dollars are spent every year to provide care to patients with these often difficult-to-heal and recurrent chronic wounds. Regardless of the etiology of chronic wounds, their treatment is usually a difficult, long-lasting process with a serious risk of microbial invasion that can lead to serious complications, such as slow healing, pain, extension of wound size, and systemic illness [4]. Furthermore, patients with chronic leg ulcers are exposed to many risk factors for antibiotic resistance. Pathogens present in chronic wounds (e.g., *Staphylococcus aureus* and *Pseudomonas aeruginosa*) have evolved to resist new antibiotics, primarily by the modification of their molecular pathways. Therefore, it is imperative to develop novel approaches for overcoming antibiotic-resistant infections and to minimize the risk of the expansion of multi-drug-resistant microorganisms [5,6,11]. Several light-based strategies purposely designed to overcome microbial resistance have been developed. Among them, photodynamic inactivation (PDI) is probably the most promising in the treatment of infectious diseases because photogenerated ROS can lead to complete eradication of a variety of resistant bacteria strains. PDI is also referred to as antimicrobial photodynamic therapy (A-PDT) and photodynamic antimicrobial chemotherapy (PACT). It is generally recognized that it may become a next-generation therapy for the treatment of oncological, as well as infectious, diseases [12,13,14,15,16]. A-PDT seems to be an important alternative to antibiotics. A-PDT causes non-selective damage to different organelles in microbes and photodynamic-resistant bacterial strains have rarely been reported [17,18,19,20,21]. Due to the problem of antibiotic resistance, photodynamic therapy (PDT) is being developed as a novel antimicrobial treatment. In a study by Morley et al., light-activated cationic photosensitizer PPA904 (3,7-bis(N,N-dibutylamino) phenothiazin-5-ium bromide) was used in sixteen patients with chronic leg ulcers and sixteen patients with diabetic foot ulcers, in a blinded, randomized, placebo-controlled, single-treatment, Phase IIa trial. All patients had an ulcer duration of >3 months, bacterially colonized with >10 colony-forming units per cm. Treatment was well tolerated and this first controlled study of PDT in chronic wounds demonstrated significant reduction in bacterial load and a trend towards wound healing was observed [19].

In our study, we performed ALA-PDT in 10 patients with chronic leg ulcers, and at 8-month follow-up, complete remission (CR) was obtained in four patients (40%), partial response (>50% reduction in ulcer diameter) in four patients (30%), and no response in two patients (10%), and these results confirm Morley’s observation about the potential of A-PDT as an alternative to antibiotics [19]. Lin et al. reported success of aminolevulinic acid-mediated A-PDT to enhance wound healing of chronic ulcers for three patients who were obstinate to usual treatments. These ulcers healed after one to three sessions of A-PDT and there was no recurrence for more than 29 months. Lin stressed that A-PDT may be an effective treatment for patients with recalcitrant infected ulcers [17]. Cappugi described the treatment of 19 patients with refractory chronic venous ulcers using photodynamic therapy. The ulcers healed in 15 cases (78.9%) after an average of 6.8 photodynamic therapy sessions (range 6.0–8.0). In the remaining four cases, the ulcers showed marked improvement after 10 photodynamic therapy sessions. The author had the same conclusion from the study as ours, namely that photodynamic therapy seems to represent a good alternative therapeutic choice for refractory chronic venous ulcers [21]. Monami et al. performed A-PDT in patients with clinically infected ulcers who had been treated with RLP068 and demonstrated that the photosensitizer RLP068 under illumination seems to be a promising topical wound management procedure for the treatment of infected diabetic foot ulcers [22]. What process may underlie tissue healing after PDT? Corsi et al. performed a pilot study to assess the microscopical parameters in skin ulcers caused by chronic venous insufficiency of the lower extremities (i.e., chronic leg ulcers) in 15 patients refractory to previous conventional treatments during photodynamic therapy (PDT). After achievement of PDT, fibroblasts appeared to further increase in number. The authors suggested that fibroblasts play an essential role in the wound healing process upon PDT treatment, given their early and intense reaction to injury [23]. A novel phthalocyanine-derived photosensitizer used for controlling bacterial load in different leg ulcers showed that PDT was effective in reducing bacterial load after the first treatment, and after the second PDT session, bacterial swab results were negative in all but two ulcers. The procedure was well tolerated in all but four patients, who reported very severe pain at baseline, which increased during treatment [18]. Aspiroz et al. presented a study with the use of methylene blue PDT in two cases of chronic lower limb ulcers in which fungal and bacterial superinfection complicated management. The authors revealed that PDT with methylene blue is a valid option for the management of superinfected chronic ulcers, reducing the use of antibiotics and the induction of resistance [20]. On the basis of results obtained in our study and the results of previous studies, it seems that A-PDT, due to its mechanism of action, low invasiveness, and lack of significant side effects, offers a potential alternative for the treatment leg ulcers.

## 4. Materials and Methods

The aim of the study was to determine the effectiveness of photodynamic therapy in the treatment of ulcers in a selected group of patients.The study was randomized.

Twenty patients from the Department of Internal Diseases, Angiology, and Physical Medicine were recruited. The patients were randomized alternatively into two experimental groups: the first receiving 5-aminolevulinic acid photodynamic therapy (ALA-PDT) (10 patients), and the second control group of 10 patients receiving local octenidine dihydrochloride (octenilin gel) and placebo red light. The procedure was performed in both groups, using protective glasses and an additional layer of cotton gloves to prevent them from seeing the procedure being carried out.

Exclusion was held consecutively to the scheduling of patients, regardless of the randomization list.

The criteria that excluded or included patients in our study were as follows:

Exclusion criteria:

- advanced atherosclerotic changes requiring angiosurgical treatment (color Doppler ultrasounds, 3D computed tomography angiography—angiogram);
- cancer;
- severe debilitating diseases;
- severe respiratory and cardiovascular diseases;
- venous thromboembolism;
- increased side effects of therapy;
- presence of hypersensitivity or allergy to any of the substances under study;
- pregnancy.

**Figure 10:**
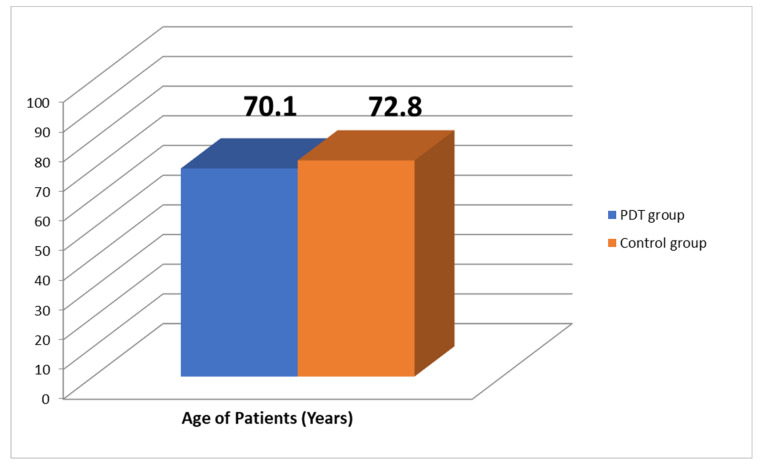
Age of patients.

Inclusion criteria:

- patients of both sexes aged 40–85 years (Figure 10), with chronic leg ulcers, who had not responded well to conventional treatment;
- no use of topical pharmaceutical products or systemic drugs (antibiotics) for at least 4 weeks.

### 4.1. Diagnostics

The research was carried out in the Department of Internal Diseases, Angiology and Physical Medicine, and the Centre for Laser Diagnostics and Therapy. Prior to treatment, a blood assessment, swab for bacteriological examination, chest X-ray, abdominal ultrasound and color Doppler ultrasounds, and 3D computed tomography angiography angiograms of lower extremity vessels were performed.

In the next step, ulcer tissue fluorescence was assessed by a CCD-based imaging system. For leg ulcer diagnosis, we used an autofluorescence diagnostic-based Onco-LIFE light source (Xillix Technologies Corp., Richmond, BC, Canada, excitation wavelength 442 nm). Tissue accumulation of Protoporphyrin IX (PpIX) showed red fluorescence (Figure 5 and Figure 6). Autofluorescence diagnostics is based on the observation of tissue fluorescence which occurs as a result of irradiation of tissue with a specific wavelength. We used the Onco-LIFE camera in conjunction with the Onco-LIFE camera controller. The light source characters include dual-mode operation for white light and fluorescence tissue imaging with a 150 W super-high-pressure mercury (Hg) arc main lamp with a halogen backup lamp. The red and green wavelengths of the autofluorescence image are filtered and amplified by image-intensifying cameras. The images are analyzed and are presented as a single real-time image on a monitor. Researchers have proposed algorithms that increase the effectiveness of detecting abnormal tissue, especially dysplastic and neoplastic, but also inflammed tissue in pulmonology, gastrology, and urology [24,25]. High-fluorescence fields are interlaced with low-fluorescence areas that are close to normal, or clearly lower, and correlated with the NCV index [26,27,28,29].

### 4.2. Drug Application

**Figure 11:**
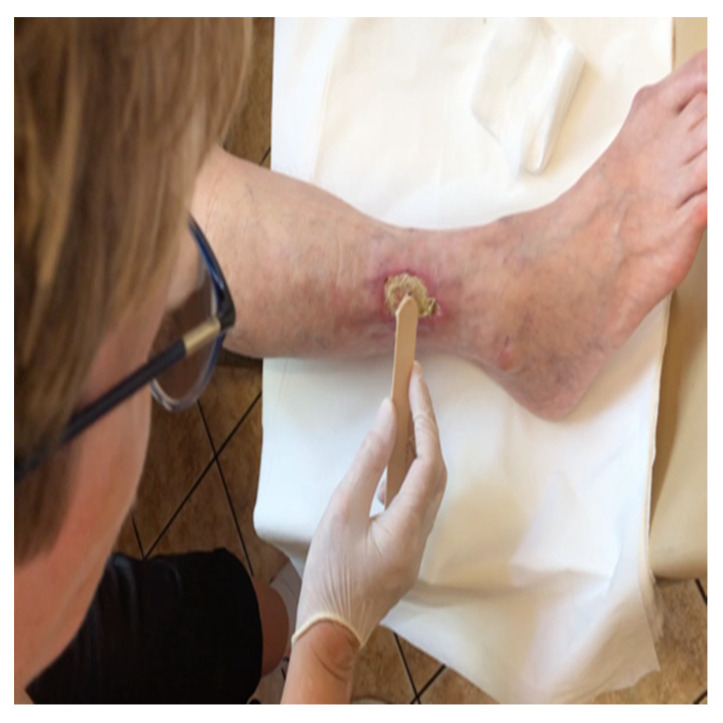
5-ALA topical application.

In our study, 5-aminolevulinic acid (ALA) (Medac GmbH, Wedel, Germany) was used as a precursor in the biosynthesis of heme. A thin layer of an oil-in-water emulsion containing 20% ALA was applied topically to the ulcer with a margin of 4 mm of surrounding normal tissue (Figure 11). Before application of the solution, the skin was cleansed with a 0.9% NaCl solution. After applications of 20% ALA or octenilin gel on the treatment area, the lesions were covered with an occlusive polyethylene film for the 4 h incubation period. A cotton glove was worn to block ambient light.

### 4.3. PDT Procedure

During the 14-day hospitalization, 10 PDT cycles were performed. Patients were hospitalized during treatments to avoid light-related adverse events.

**Figure 12:**
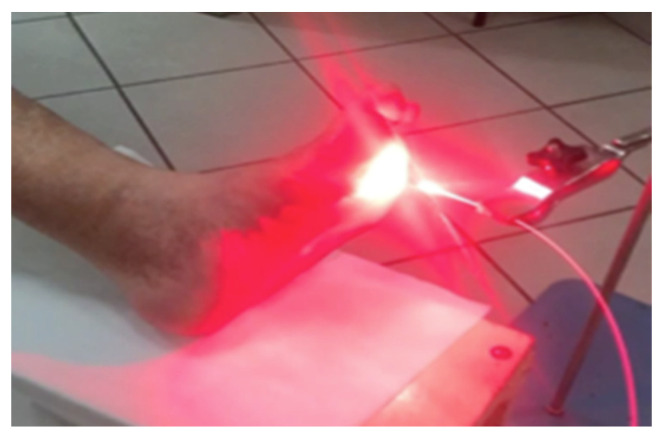
The PDT procedure.

Irradiation in the PDT group was performed using 630 nm laser light from a Diomed 630 with a radiation dose of 80 J/cm^2^ at a distance of five to eight centimeters (Figure 12). We included at least a 10–20% margin around the lesions in the field of irradiation. The treatment time was around 15 min at a low fluence rate that was interrupted periodically. All of the patients received local anaesthesia (10% lidocaine hydrochloride) and 20% of patients were given analgesic drugs (i.e., paracetamol).

All patients wore safety glasses and cotton gloves to block ambient light.

The control group of 10 patients receiving local octenidine dihydrochloride (Octenilin gel) were irradiated using a placebo light source with an inserted filter that mimicked red light.

### 4.4. Follow-Up Study

Our study is a pilot study, and the post-treatment observation period was 8 months. Complete responses were judged as the clinical absence of an ulcer, which corresponded to green fluorescence of healthy tissue. A partial response was defined as a reduction of half (>50% reduction in ulcer diameter) in the lesion area. There were nonresponders that had no response after 8 months and 5 sessions of 10 PDT.

### 4.5. Statistics

Student’s *t*-test was used for data comparison and *p* values < 0.05 were considered to be statistically significant.

## 5. Conclusions

Our preliminary observations showed that PDT can be used to treat leg ulcers as a minimally invasive and effective method with no serious side effects. It seems that the following mechanisms of action of photodynamic theotherapy lie at the heart of this process: antimicrobial effect, modulation of inflammatory process, and improving the oxygenation of ulcer tissue, resulting in a reduction in healing time and improved scarring results [30,31].The treatment is generally well tolerated; the side effects and tolerance of PDT are associated with wound size and degree of wound infection. Further studies on a larger group of patients are needed to confirm the beneficial role of PDT in the treatment of LUs and in potential applications of pH-responsive nanomaterials in anti-infective therapy [32]. Due to the significant medical and social problem of chronic leg ulcers, attempting to use photodynamic therapy for their treatment is an advantage, especially in regard to the frequent use of chronic antibiotic therapy, which results in antibiotic resistance.

## Figures and Tables

**Table 1:** Differentiation of venous, ischemic and neuropathic ulcers.

Ulcers Types	Venous	Ischemic	Neuropathic
**Gender**	More often women	More often men	Women/men
**Interview**	History of thrombophlebitis	overweight, high blood pressure, smoking, diabetes	diabetes
**Localisation**	medial, lateral or on the back of the calf, above the ankles	toes, pressure points, medial edge of the heel, edge of the foot, dorsal side of the toes	sole, bone prominences, often under the callus
**Appearance**	thick cylindrical wound edge, pink base, exudate	irregular edges, white/blue, visible tendons or bones, weak granulation tissue	irregular, indented edges, red granulation, deep, infected, often visible deeper structures
**Exudation**	intense yellow-pink discharge, pus	little or no exudate	medium oozing
**Foot warmth**	warm	cool, dry	warm, humid
**Pain**	Medium when standing	medium, when standing, disappears when the limb is lifted	absent
**Puls**	present	absent	Present or absent
**Veins**	varicose veins, telangiectasias	collapsed veins	Dilated veins
**Feel**	present	variables	absent
**Ulceration** **in the calluses**	absent	rare	present

**Table 2:** Two groups of patients (PDT/Control) with Lus.

Group	No.	Age	Wound Area before [cm^2^]	Wound Area after [cm^2^]	Complete Response (CR)	Partial Response (PR)	No Response (NR)	Reducing Bacterial Load *		Etiology **	Numeric Pain Rating Scale (NPRS)	Side Effect: Edema	Side Effects: Swelling Erythema Inflamation
**PDT**	1	72	49	9	0	1	0	4	3	CVI + PAD	7	1	0
**PDT**	2	84	25	0	1	0	0	4	2	CVI + PAD	6	0	0
**PDT**	3	78	9	0	1	0	0	3	1	CVI	4	0	0
**PDT**	4	82	20	20	0	0	1	3	3	CVI + PAD	10	1	1
**PDT**	5	67	4	0	1	0	0	2	0	NF	2	0	0
**PDT**	6	70	9	2	0	1	0	3	2	CVI	3	0	0
**PDT**	7	58	2.25	0	1	0	0	2	0	NF	1	0	0
**PDT**	8	60	18	6	0	1	0	3	2	CVI	5	0	0
**PDT**	9	54	20	8	0	1	0	4	3	CVI + PAD	6	0	0
**PDT**	10	76	16	16	0	0	1	3	3	CVI	10	1	1
	**Sum**				4	4	2				5.5	3	2
	**Average**	70.1	17.225	6.1									
**Control**	1	68	9	2	0	1	0	3	3	CVI + PAD	4	0	0
**Control**	2	81	16	18	0	0	1	4	2	CVI + PAD	5	1	0
**Control**	3	70	3	0	1	0	0	2	1	NF	5	0	0
**Control**	4	56	21	3	0	1	0	4	2	CVI + PAD	1	0	0
**Control**	5	77	24	24	0	0	1	4	4	CVI + PAD	2	0	0
**Control**	6	71	18	0	1	0	0	4	4	CVI	4	1	1
**Control**	7	82	12	15	0	0	1	3	2	CVI	3	0	0
**Control**	8	83	15	12	0	0	1	3	2	CVI	3	0	0
**Control**	9	74	18	12	0	0	1	2	2	CVI + PAD	6	0	0
**Control**	10	66	45	15	0	1	0	4	4	CVI	7	0	0
	**Sum**				2	3	5				3.2	2	1
	**Average**	72.8	18.1	10.1									

* Reducing bacterial load: 4: ++++; 3: +++; 2: ++; 1: +; All the wounds contained polymicrobial flora, especially *Staphylococcus aureus*, whereas *Pseudomonas aeruginosa*, *Enterobacteriaceae*, *Streptococcus pyogenes*, Most common: *Bacteroides gr. fragilis*, *Peptostreptococcus*, *Fusobacterium*, *Corynebacterium* spp and *Candida* spp. ** Chronic venous insufficiency (CVI), peripheral arterial disease (PAD), neuropathic foot (NF), combined occurrence of chronic venous insufficiency and peripheral arterial disease (CVI + PAD).

## Data Availability

The data presented in this study are available on request from the corresponding author.
